# Relational Consensus and Gendered Differentiation: The Ranked Structure of Ideal Partner Preferences Among Chinese University Students

**DOI:** 10.3390/bs16101921

**Published:** 2026-10-16

**Authors:** Zhonglu Li, Jiahui Yu, Yuanyuan Hu

**Affiliations:** School of Philosophy and Social Development, Shandong University, Jinan 250100, China; sozlli@sdu.edu.cn (Z.L.); yujiahui@mail.sdu.edu.cn (J.Y.)

**Keywords:** ideal partner preferences, values, partner evaluation, gender-role attitudes, romantic relationships, gender differences, ranked choice, China

## Abstract

Relational qualities are highly valued in ideal partners, while women and men differ in the weight they attach to resources and physical attractiveness. It remains unclear whether these patterns reflect competing ideals or different emphases within a broadly shared preference hierarchy. Using top-three partner criteria reported by 4704 undergraduates from 12 Chinese universities, this study examines how desirable partner characteristics are ordered when respondents must prioritize among multiple options. Exploded rank-ordered logit models showed that relationship-sustaining qualities accounted for 82.4% of first choices and remained the most common domain at the second and third ranks. Status-security and eligibility-related attributes appeared more often at later ranks. Relative to women, men were less likely to prioritize status-security attributes and more likely to prioritize eligibility-related attributes. Higher family socioeconomic status was associated with greater relative emphasis on status-security attributes, whereas university tier showed no independent association after adjustment. More egalitarian gender-role attitudes and current romantic involvement were associated with greater emphasis on relationship-sustaining qualities. The results indicate substantial agreement in the highest-ranked relational qualities, alongside gendered and socially patterned differences in the weighting of other partner characteristics.

## 1. Introduction

Warmth, trustworthiness, kindness, and mutual understanding are commonly rated among the most desirable qualities of a long-term partner (Fletcher et al., 1999; Sprecher & Regan, 2002; Valentine et al., 2020). Women also tend to place greater importance on a partner’s economic prospects, whereas men tend to place greater importance on physical attractiveness (Buss et al., 2001; Buss & Schmitt, 2019; Thomas et al., 2020). These gender differences have persisted even as the importance attached to particular partner characteristics has changed over time (Buss et al., 2001; Buss & Schmitt, 2019; Thomas et al., 2020). What remains unclear is whether gender differences reflect distinct structures of partner ideals or different emphases within a broadly shared preference hierarchy. Empirically, this distinction turns on whether gender differentiation extends to the qualities placed at the top of the ranking or is concentrated elsewhere in the ordering.

Separate importance ratings cannot fully answer this question. Respondents may regard shared values, kindness, health, education, income, and attractiveness as important without assigning them equal priority when these qualities are considered together. Independent ratings identify which characteristics are valued but provide less information about their relative position when several desirable qualities must be prioritized (Fletcher et al., 1999; Valentine et al., 2020). Constrained preference tasks address this problem by requiring trade-offs among desirable characteristics (Li et al., 2002; Simons et al., 2025; Thomas et al., 2020). Ranked choices add an ordinal dimension by showing which characteristics are placed ahead of others. Group differences may therefore concern not whether a characteristic is valued, but where it is located within the ordering of desirable partner qualities.

Partner ideals also have an evaluative dimension that connects mate-preference research to the study of values. Classical accounts describe values as standards for judging what is desirable and for ordering competing goals or alternatives (Allport et al., 1960; Rokeach, 1973; Feather, 1992). In the present study, this literature provides a language for understanding ranking as evaluative ordering; it does not imply that the 17 partner characteristics constitute a general values scale. The empirical focus remains the ordered structure of partner preferences: which relational, material, physical, and social considerations respondents place ahead of others when they cannot assign every desirable quality the same priority.

Ranked ideals should not be treated as fixed decision rules for attraction, dating behavior, or eventual partner choice. They are prospective statements of preference, and some qualities may be easier to identify and articulate in advance than others. Shared values, responsibility, and personality provide familiar ways of describing a desirable long-term partner, whereas physical attraction may depend more strongly on a particular person and on interaction as it unfolds. The relevance of stated ideals also depends on the partners available and the circumstances under which judgments are made (Gerlach et al., 2019; Eastwick et al., 2023). Rankings therefore capture the explicit ordering of stated partner criteria rather than the psychological weight those criteria will necessarily carry in an actual encounter.

This distinction is particularly important for gender. Women and men may agree on the relationship-sustaining qualities they value most, including shared values and ideals, personality, responsibility, health, shared interests, and lifestyle habits, while assigning different priority to other partner characteristics. Women’s greater emphasis on economic prospects may be connected to expectations surrounding financial provision, housing, childrearing, and the economic consequences of marriage. Men’s greater emphasis on appearance may reflect gendered standards of desirability in partner evaluation (Buss & Schmitt, 2019; Yu et al., 2026). Independent ratings establish average differences in the importance assigned to particular characteristics, but they do not show where those differences are located within the preference hierarchy.

Family background may shape this hierarchy in a different way. Family resources affect access to education, financial support, housing assistance, and social networks. Students from advantaged families may place greater priority on a partner’s education and economic prospects because these characteristics help preserve expected living standards or maintain social boundaries. Yet family security may reduce dependence on a partner’s resources and permit greater emphasis on relationship-sustaining qualities. Among Chinese university students, family background is associated with attitudes toward love, although the direction and magnitude of that association vary across measures and populations (Hu & Wu, 2019; Tang & Wu, 2026). University context may matter for similar reasons. Institutions differ in selectivity, peer composition, career prospects, and the value attached to educational credentials, all of which may affect the relative position of status-related partner characteristics.

Romantic involvement and gender-role attitudes introduce two further sources of variation in partner evaluation. Relationship experience can make communication, mutual adjustment, and everyday compatibility more concrete. Gender-role attitudes, by contrast, represent individual orientations toward the gendered organization of paid work, economic provision, housework, childcare, and family responsibility. Social-role accounts propose that expectations about women and men are partly shaped by their positions in the division of labor and by the social arrangements surrounding paid and unpaid work (Eagly & Wood, 1999). Respondent gender and gender-role attitudes therefore need not capture the same source of variation in partner evaluation. A student may occupy the same gender category as another while holding quite different views about provision, care, reciprocity, and the division of family responsibilities.

Chinese undergraduates occupy a position in which these considerations are likely to intersect. They are gaining autonomy in education, employment, and intimate life while often remaining economically dependent on their families. Parents commonly finance university education and may later contribute to housing and family formation. At the same time, young adults form relationships within a social context in which expectations surrounding economic provision and care remain unevenly distributed between women and men (Hu & Wu, 2019; Yeung & Hu, 2016). Relationship compatibility, family resources, and future economic security can therefore matter simultaneously in judgments about a desirable long-term partner.

Studies in China show that women and men respond to many of the same desirable partner characteristics but assign different weights to some of them. Women tend to place greater importance on education, income, and property, whereas men place relatively greater weight on appearance (Xu, 2025; Yu et al., 2026). Preferences also vary with education, family background, rural or urban origin, age, and romantic experience (Hu & Wu, 2019; Tang & Wu, 2026; Yu et al., 2026). Profile-based survey experiments, including conjoint or vignette designs, estimate trade-offs among attributes jointly presented within hypothetical partner profiles, whereas conventional questionnaire surveys more often ask respondents to rate or select partner characteristics individually. Neither design directly identifies where group differences fall within an ordered preference hierarchy.

The present study examines this issue using top-three rankings of partner characteristics reported by 4704 undergraduates from 12 Chinese universities in the 2020 Panel Study of Chinese University Students (PSCUS). Respondents selected and ordered three characteristics from a survey-provided set of 17. For analysis, the characteristics were organized into three domains: relationship-sustaining qualities, status-security attributes, and eligibility-related attributes. These domains are analytical groupings rather than validated psychological scales. The Ideal Standards Model provides useful conceptual vocabulary for organizing the secondary survey items, but the study does not claim to test or extend its original dimensions (Fletcher et al., 1999; Campbell & Fletcher, 2015). In the present study, the eligibility-related category is an analytical grouping rather than an ISM dimension, bringing together appearance, age, romantic history, ethnicity, and region. These characteristics have been examined separately in prior mate-preference research (Buss et al., 2001; Chen et al., 2015; Yu et al., 2026). Domain-level findings are therefore interpreted alongside item-level patterns and sensitivity analyses rather than as evidence for three underlying psychological dimensions.

Taken together, these considerations direct attention to where consensus and differentiation occur within the ranking, rather than simply whether women and men value different partner characteristics. The central empirical contribution of the study is to examine whether substantial agreement about the qualities placed at the top of the ordering can coexist with gendered and socially patterned differentiation elsewhere in the same preference structure. Directional expectations and open questions are specified below.

### 1.1. Values and Priorities Under Constraint

The Ideal Standards Model (ISM) distinguishes three broad dimensions of partner ideals: warmth-trustworthiness, vitality-attractiveness, and status-resources (Fletcher et al., 1999; Campbell & Fletcher, 2015). Warmth-trustworthiness concerns responsiveness, dependability, and the capacity to sustain intimacy; vitality-attractiveness concerns physical appeal and energy; and status-resources concerns competence, social position, and material prospects. These dimensions are useful here as conceptual reference points rather than as constructs directly measured by the PSCUS questionnaire. Warmth-trustworthiness predicts attraction in early encounters and satisfaction in established relationships (Valentine et al., 2020), while trade-off studies show that desirable partner characteristics can acquire different priority when considered jointly rather than independently (Fletcher et al., 2004; Li et al., 2002).

The three ISM dimensions also foreground different desirable outcomes. Warmth-trustworthiness centers mutuality, dependability, and relationship maintenance; status-resources centers economic security, competence, and social position; and vitality-attractiveness centers physical and interpersonal desirability. The present study treats these as value-laden considerations within partner evaluation, not as direct measures of general personal values. Here, values refer to the evaluative content expressed through the ordering of desirable characteristics and should not be confused with the single questionnaire item “shared values and ideals.”

Partner ideals therefore involve not only whether particular characteristics are desirable, but also how they are ordered relative to one another. When respondents must weigh warmth-trustworthiness, vitality-attractiveness, and status-resources jointly, their choices may differ from judgments made about each dimension in isolation (Fletcher et al., 2004). Ranking imposes a similar constraint in ordinal form by requiring respondents to place some characteristics ahead of others.

The PSCUS survey was not designed as a direct test of the Ideal Standards Model, and its 17 criteria do not map one-to-one onto the three ISM dimensions. We therefore used a theory-guided, content-based classification rather than treating the items as direct indicators of ISM dimensions. Items were assigned according to their primary substantive content: criteria centered on interpersonal compatibility and long-term relationship maintenance were classified as relationship-sustaining; criteria centered on socioeconomic position and material security as status-security; and personal, demographic, and background characteristics relevant to prospective partner suitability but not primarily relational or socioeconomic as eligibility-related. Relationship-sustaining qualities include shared values and ideals, personality and temperament, responsibility, health, shared interests, and lifestyle habits. Status-security attributes include family origin and social ties, education, occupation, housing, income, and assets. Eligibility-related attributes include appearance and bearing, age, romantic history, ethnicity, and region.

These domains are analytical categories rather than psychological scales. The eligibility-related category is especially heterogeneous: appearance, age, romantic history, ethnicity, and region need not express a single underlying psychological construct. Domain-level results are therefore interpreted alongside item-level patterns. Health likewise permits more than one interpretation. We place it with relationship-sustaining qualities in the primary analysis because the questionnaire lists health separately from appearance and bearing and because general health may bear on long-term functioning and relationship continuity. At the same time, health may also function as a screening characteristic or as information related to physical desirability. We therefore examine an alternative classification in the sensitivity analyses rather than treating its primary placement as the only theoretically plausible one.

Rank position provides the relevant comparison. The first criterion is chosen from all 17 alternatives, whereas the second and third are chosen from the alternatives remaining after earlier selections. A domain that appears more often at the second or third rank has not become more important in absolute terms. Rather, its attributes account for a larger share of the remaining choices after higher-ranked characteristics have been removed.

### 1.2. Gender Differences Across the Preference Hierarchy

Women and men differ most consistently in the relative weight they assign to economic resources and physical attractiveness. Women tend to place greater weight on a prospective partner’s economic prospects, whereas men place greater weight on physical attractiveness (Buss et al., 2001; Buss & Schmitt, 2019; Thomas et al., 2020). A similar pattern is evident in China, although both women and men respond favorably to a broad range of desirable partner characteristics (Blair & Madigan, 2016; Chen et al., 2015; Yu et al., 2026).

These differences need not extend to the characteristics placed at the top of the ranking. Shared values and ideals, personality, responsibility, and other relationship-sustaining qualities may occupy similarly high positions for women and men, while economic and appearance-related characteristics receive different relative weights. An ordered preference structure therefore allows substantial agreement over relationship-sustaining qualities to coexist with differentiation in other criteria of partner suitability.

Status-security attributes may carry greater relative weight for women when partnership is evaluated within a social context where economic provision, employment interruption, childbirth, and care responsibilities remain gendered. Education, occupation, income, housing, and assets can bear directly on anticipated economic security under these arrangements. Appearance and age may carry greater relative weight for men within prevailing standards of partner desirability (Buss & Schmitt, 2019; Yu et al., 2026). These are contextual explanations for aggregate gender differences between women and men as respondents. Respondent gender was observed, whereas target-partner gender was not: the survey did not measure sexual orientation or ask respondents to identify the gender of the partner they had in mind. Accordingly, the analysis estimates differences between self-reported women and men without assuming the gender of the preferred partner.

### 1.3. Family Resources, University Context, and Relationship Orientations

Family background may shape the weight assigned to socioeconomic characteristics in partner preferences. Students from more advantaged families may place greater emphasis on education, occupation, income, and other status-related attributes when expectations about partnership are tied to maintaining living standards or social boundaries. Among Chinese university students, higher parental education and family advantage have been associated with more realistic attitudes toward love (Hu & Wu, 2019; Tang & Wu, 2026). Family background also remains relevant to marital matching and stratification in China (Dong & Xie, 2023; Liu et al., 2024). At the same time, parental resources may provide financial support and housing security that reduce reliance on a partner’s economic position. Family advantage may therefore either heighten expectations of socioeconomic compatibility or lessen the need to obtain security through a partner.

University tier reflects a different dimension of social position. Students of different social origins follow different educational and post-college trajectories even within elite Chinese universities (Niu et al., 2020), while elite-university attendance is associated with advantages in early labor-market outcomes after prior academic achievement is taken into account (Démurger et al., 2024). University prestige may therefore shape expectations about education, occupation, and future economic position independently of family background.

The implications of university tier for partner preferences are not straightforward. Students in elite universities may place greater value on educational and occupational compatibility because they anticipate relatively advantaged trajectories of their own. Stronger educational and career prospects may also reduce the need to seek socioeconomic security through a partner. Family socioeconomic status and university tier are therefore examined as separate correlates of partner-preference ordering.

Gender-role attitudes concern how individuals believe paid work, economic provision, housework, childcare, and family responsibilities should be organized between women and men. They can be understood as individual orientations toward broader gendered social arrangements rather than as a proxy for respondent gender itself. Social-role theory links gender-differentiated expectations to the division of labor and relative social positions of women and men (Eagly & Wood, 1999). Such attitudes are also socially transmitted and associated with later educational, employment, and family-role outcomes (Dhar et al., 2019; Johnston et al., 2014). In mate selection, Eastwick et al. (2006) found that stronger endorsement of traditional gender ideology was associated with more sex-typed partner preferences across nine nations.

The relevance of gender-role attitudes becomes particularly apparent when partner characteristics must be prioritized. Using rank-ordered mate preferences, Rempala et al. (2014) found that traditional men placed greater priority on physical attractiveness than egalitarian men, whereas traditional women placed greater priority on social standing and less on kindness. Cross-national evidence also suggests that gender differentiation in mate preferences is neither wholly invariant nor reducible to a single national-level explanation: some research reports smaller sex differences where gender parity is greater (Zentner & Mitura, 2012), while substantial gender differences are also observed across diverse cultural settings (Buss, 1989; Thomas et al., 2020). This makes it useful to distinguish respondent gender from the respondent’s own orientation toward gendered provision, care, and reciprocity. More egalitarian attitudes should be associated with greater relative emphasis on relationship-sustaining qualities, but whether that association differs between women and men is less clear.

Current romantic involvement has no equally clear directional implication. Experience within a relationship may make communication, compromise, and everyday compatibility more salient, but it may also make financial obligations and other practical demands of partnership more concrete. Tang and Wu (2026) found that romantic experience altered the association between family background and attitudes toward love among Chinese university students rather than producing a uniform shift in attitudes. The cross-sectional design also cannot distinguish preferences shaped by relationship experience from preferences associated with entering or maintaining a relationship.

### 1.4. Research Questions and Hypotheses

The preceding literature provides a broader empirical framework for questions concerning family socioeconomic status, university context, romantic involvement, and gender moderation, while supporting directional expectations for the overall rank structure, gender differences, and gender-role egalitarianism. Accordingly, the research questions are presented first, followed by the more specific directional hypotheses.

**Research** **Question** **1.** *How are family socioeconomic status and elite-university attendance associated with preference-domain priorities?*

**Research** **Question** **2.** *Does the association between gender-role egalitarianism and preference-domain priorities differ between women and men?*

**Research** **Question** **3.** *How is current romantic involvement associated with preference-domain priorities?*

**Hypothesis** **1.** *Relationship-sustaining qualities will be more likely than status-security and eligibility-related attributes to occupy the first rank.*

**Hypothesis** **2.** *Relative to women, men will place less emphasis on status-security attributes and greater emphasis on eligibility-related attributes, relative to relationship-sustaining qualities.*

**Hypothesis** **3.** *More egalitarian gender-role attitudes will be associated with greater relative priority for relationship-sustaining qualities than for status-security and eligibility-related attributes.*

## 2. Materials and Methods

### 2.1. Data and Analytic Sample

Data come from the 2020 Panel Study of Chinese University Students (PSCUS), conducted by the Institute of Sociology at the Chinese Academy of Social Sciences. The survey used a multistage stratified design covering higher education institutions, academic units, and classes. It obtained 18,405 valid responses from postgraduate, undergraduate, and vocational students across 21 institutions. This study focuses on 9459 undergraduates enrolled in 12 universities. Eight of the universities had historically been designated under Project 985 or Project 211, national initiatives that concentrated central support in a selected group of high-prestige universities; four were ordinary undergraduate universities. This historical designation is used below to distinguish the two university tiers.

The partner-ranking item appeared on one of two questionnaire forms assigned randomly by the survey. Of the 9459 undergraduates, 4795 received the form containing the ranking item and 4664 received the alternative form. A balance model based on the 9284 respondents with complete data on five observed characteristics showed no systematic difference in assignment by gender, year of study, precollege residence, field of study, or family socioeconomic status; the joint test was nonsignificant, *p* = 0.848 (Supplementary Table S1).

All 4795 respondents who received the ranking item provided three valid, nonduplicate choices. Ninety-one had missing data on at least one covariate, yielding a complete-case analytic sample of 4704. No values were imputed, and all primary models use the same analytic sample. Participation in the source survey was voluntary and anonymous, and the analyses use de-identified secondary data.

### 2.2. Measures

#### 2.2.1. Ranked Ideal Partner Preferences

Respondents were asked, “When choosing a marriage partner, which conditions do you consider relatively important? Please select three items from the following options and rank them in order of importance.” The 17 response options were family origin and social ties, education, occupation, shared values and ideals, appearance and bearing, housing, income, health, personality and temperament, shared interests, lifestyle habits, romantic history, ethnicity, region, responsibility, assets, and age. The 17-item pool was specified in the 2020 PSCUS questionnaire rather than constructed for the present study. The questionnaire does not identify a separate published scale-development source for these criteria. We therefore treat the 17 items as survey-provided partner criteria rather than as a validated inventory. The items cover characteristics commonly examined in international and Chinese mate-preference research, including personality and values, physical attractiveness, socioeconomic resources, education, family background, and responsibility (Buss et al., 2001; Fletcher et al., 1999; Chen et al., 2015; Yu et al., 2026).

The 17 criteria were grouped into the three analytical domains defined above. The classification was theory-guided and content-based rather than derived from factor analysis or another data-driven procedure. Each criterion was assigned to one domain according to its primary substantive content. Relationship-sustaining qualities comprised shared values and ideals, personality and temperament, responsibility, health, shared interests, and lifestyle habits. Status-security attributes comprised family origin and social ties, education, occupation, housing, income, and assets. Eligibility-related attributes comprised appearance and bearing, romantic history, ethnicity, region, and age. These mutually exclusive categories organize a ranked-choice task for comparative analysis; they are not reflective multi-item psychological scales. Because respondents selected three nonredundant criteria and the grouped items were not designed as indicators of a common latent construct, internal-consistency coefficients such as Cronbach’s alpha are not appropriate for evaluating these domains. Classification robustness is instead examined through item-level analyses, a specification excluding ethnicity and region, and a sensitivity analysis that reclassifies health as eligibility-related.

#### 2.2.2. Family Socioeconomic Status and University Tier

Family socioeconomic status was measured by the first principal component of four indicators: the higher educational attainment reported for either parent, the higher parental occupational-position score, the natural logarithm of parental monthly income, and the respondent’s assessment of the family’s relative economic standing. Parental education categories were converted to approximate years of schooling (0, 6, 9, 12, 15, 16, or 19 years), and the higher value for either parent was retained. Parental occupation was coded on a six-point ordered score, from 1 for unemployment to 6 for senior leadership, with intermediate scores distinguishing manual and agricultural work, self-employment, and professional or managerial positions; again, the higher parental score was retained. Reported monthly parental income was represented by the midpoint of the relevant income bracket (RMB 250 to RMB 60,000) and then natural-log transformed. Relative family economic standing was coded from 1 to 5 so that higher values indicated greater advantage. The first principal component of these four indicators was oriented so that higher scores indicated greater socioeconomic advantage and then standardized (M = −0.001, SD = 0.992 in the analytic sample). Sensitivity analyses replaced this measure with three alternatives: a standardized mean of the four indicators, an objective index excluding subjective economic standing, and an index excluding parental occupation.

University tier was coded 1 for the eight universities historically designated as Project 985 or Project 211 institutions and 0 for the four ordinary undergraduate universities. Family socioeconomic status and university tier were entered separately in all relevant models.

#### 2.2.3. Gender, Gender-Role Attitudes, Romantic Involvement, and Covariates

Gender was recorded as woman or man, with men coded 1 and women 0. The questionnaire did not measure nonbinary gender identities, sexual orientation, or the gender of the desired partner. Accordingly, the analyses compare respondents who self-reported as women and men, irrespective of sexual orientation or the gender of the partner they had in mind.

Gender-role egalitarianism was measured with six statements about paid work, economic provision, housework, childcare, and family responsibility: men are better suited to earning income and women to caring for the family; it is natural for both spouses to work; spouses should share housework and childcare; when the husband’s income is sufficient after children are born, the wife should spend more time caring for the children; when work and family conflict, the wife should prioritize the family; and, for women, doing well at work is less important than marrying well. The six items cover two commonly studied components of gender-role attitudes: paid work and economic provision, and the allocation of domestic and care responsibilities (Eagly & Wood, 1999; Dhar et al., 2019; Johnston et al., 2014). Responses ranged from 1 (strongly agree) to 4 (strongly disagree). Egalitarian statements were reverse-scored so that higher totals indicated more egalitarian attitudes. Complete responses to all six items were summed (range = 6–24; Cronbach’s alpha = 0.810) and standardized for multivariate analysis.

Current romantic involvement was coded 1 for respondents who reported having a romantic partner and 0 otherwise. Covariates included year of study (third year or above versus earlier years), precollege residence (county seat or city versus rural village or town), and academic field (science, engineering, agriculture, or medicine versus humanities, social sciences, arts, or management).

Table 1 presents the descriptive statistics (M, SD, minimum, and maximum) for the two continuous variables. The remaining variables are binary and coded as 0 and 1. Men comprised 46.5% of respondents, 64.9% attended an elite university, and 26.6% were currently in a romantic relationship. Nearly half (48.4%) were in their third year or above, 56.5% studied science, engineering, agriculture, or medicine, and 64.6% had lived in a county seat or city before entering university. The mean unstandardized gender-role egalitarianism score was 19.445 on the 6–24 scale.

### 2.3. Analytic Strategy

Exploded rank-ordered logit (ERL) models represented each top-three ranking as a sequence of three conditional choices. At the first rank, a respondent selected one item from all 17 alternatives. That item was then removed, and the second choice was modeled from the 16 remaining alternatives. After the first two selections were removed, the third choice was modeled from the remaining 15. Each student therefore contributed three choice sets, with exactly one selected alternative in each set. Thus, at each rank, the model compares the selected item with the alternatives that remained available at that stage. This formulation preserves the ordering of responses and the shrinking choice set without assigning arbitrary numerical distances between first, second, and third rank.

All models included 16 item fixed effects to account for baseline differences in the likelihood that each criterion was selected. Rank-by-domain interactions captured changes across ranks in the relative likelihood of selecting status-security or eligibility-related attributes rather than relationship-sustaining qualities. Family socioeconomic status, university tier, gender, gender-role egalitarianism, romantic involvement, and the covariates entered the models through interactions with preference domain. These predictor-by-domain associations were constrained to be constant across ranks. The contextual model included family socioeconomic status and university tier; the fully adjusted model additionally included gender, gender-role egalitarianism, romantic involvement, year of study, precollege residence, and academic field. Supplementary Table S3 reports the full sequence of model specifications.

The predictor-by-domain coefficients express differences in the relative likelihood of selecting status-security or eligibility-related attributes rather than relationship-sustaining qualities. Omnibus Wald tests jointly evaluated the two domain interactions for each predictor. To facilitate interpretation of the gender differences, standardized conditional probabilities were derived from the fully adjusted model by setting gender in turn to woman and man while retaining each respondent’s observed covariates and remaining choice alternatives. Probabilities at the second and third ranks are therefore conditional on the alternatives still available at those ranks. Cluster-robust standard errors were calculated at the university-by-academic-unit level, yielding 91 clusters.

An additional model examined whether the association between gender-role egalitarianism and preference-domain priority differed by gender. This specification added gender-by-egalitarianism-by-domain interactions to the fully adjusted model, with relationship-sustaining qualities as the reference domain. Because gender and egalitarianism are respondent-level characteristics and therefore constant within each choice set, the moderation test is identified through their interactions with the status-security and eligibility-related domains. The two three-way interaction terms were evaluated jointly with a Wald test. No rank-specific moderation terms were included.

Several sensitivity and supplementary analyses assessed the robustness and interpretation of the domain-level results. First, the principal-component family SES score was replaced with three alternative constructions. Second, ethnicity and region were removed from the eligibility-related domain; respondents whose rankings included either item were excluded from this 15-item specification, leaving 4582 students. Third, to examine heterogeneity within the analytical domains, a supplementary item-specific model replaced the gender-by-domain terms with gender-by-item interactions while retaining the other components of the fully adjusted ERL. This model was used to assess whether health and other individual criteria exhibited distinct gender patterns. Finally, health was reclassified from the relationship-sustaining domain to the eligibility-related domain, and the fully adjusted model was re-estimated.

All coefficients are interpreted as associations. The cross-sectional design does not identify causal effects of family background, romantic involvement, or gender-role attitudes. Analyses were conducted in Stata 15.1 (StataCorp LLC, College Station, TX, USA).

### 2.4. Transparency and Openness

This study was not preregistered. The analyses use de-identified third-party data from PSCUS. Under the applicable data-use conditions, the authors are not permitted to publicly redistribute respondent-level data. Researchers interested in accessing the data may apply directly to the data custodian through its official procedures. The survey questionnaire is available from the data provider. The manuscript reports the sample exclusions, measures, model specifications, and sensitivity analyses used to address the research questions and hypotheses. The Stata code used for the analyses is available from the corresponding author upon reasonable request.

## 3. Results

### 3.1. Relationship-Sustaining Qualities Dominated the Ranking

**Figure 1:**
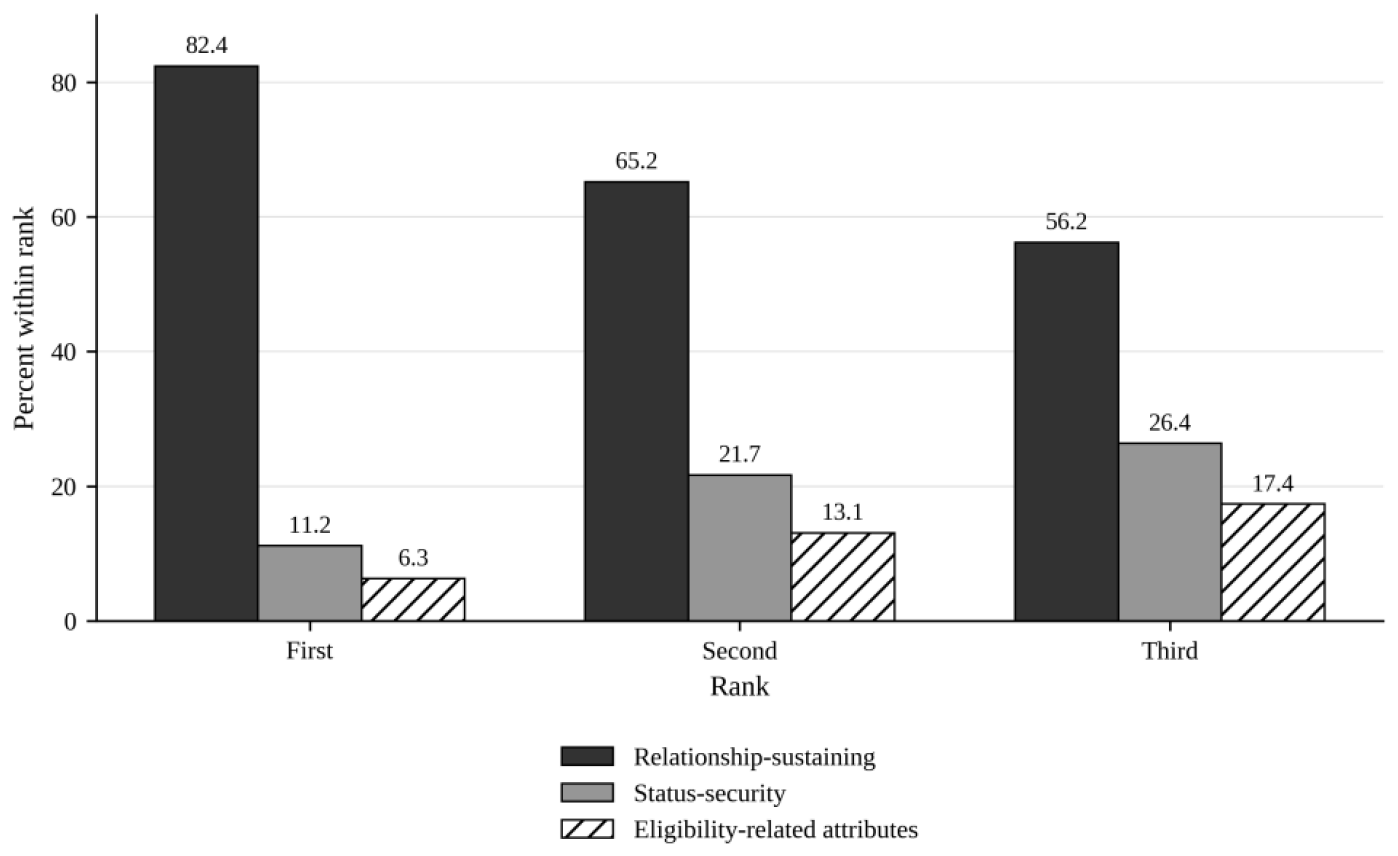
Observed distribution of preference domains by rank. Note. Percentages sum to 100 within rank. N = 4704 students.

Relationship-sustaining qualities dominated the rankings, accounting for 82.4% of first choices, 65.2% of second choices, and 56.2% of third choices (Figure 1). Status-security attributes represented 11.2% of first choices, 21.7% of second choices, and 26.4% of third choices, while eligibility-related attributes accounted for 6.3%, 13.1%, and 17.4%, respectively. The most common sequence across the three ranks was relationship-sustaining at each position (R-R-R), reported by 31.4% of students (Supplementary Table S2).

At the item level, shared values and ideals accounted for 45.4% of first choices, followed by personality and temperament at 20.7% (Table 2). Within the status-security domain, family origin and social ties was the leading first-rank item (6.7%); within the eligibility-related domain, appearance and bearing ranked highest (4.7%). Several characteristics, including appearance and bearing, family origin and social ties, income, shared interests, responsibility, and lifestyle habits, appeared more often at the second or third rank than at the first. This pattern indicates that these characteristics generally occupied lower positions in the stated preference ordering rather than being unimportant.

Table 2 also reports the mean and standard deviation of rank position among respondents who selected each characteristic; these conditional rank summaries describe where a selected item tended to appear among ranks 1–3 and do not assign ranks to unselected characteristics.

The adjusted ERL estimates confirmed the rank pattern observed descriptively (Table 3). Relative to relationship-sustaining qualities at the first rank, the log odds of selecting a status-security attribute were higher at the second rank, b = 0.608, SE = 0.059, and at the third rank, b = 0.756, SE = 0.065. The corresponding rank-by-domain coefficients for eligibility-related attributes were 0.688 (SE = 0.079) and 0.979 (SE = 0.071). The rank-by-domain terms were jointly significant, Wald χ^2^(4) = 312.082, *p* < 0.001, indicating that status-security and eligibility-related attributes occupied relatively larger shares of the remaining choices at the second and third ranks. Together with the predominance of relationship-sustaining qualities among first choices, these results support Hypothesis 1.

### 3.2. Women and Men Differed in Status-Security and Eligibility-Related Priorities

Compared with women, men had lower log odds of selecting status-security rather than relationship-sustaining attributes, b = −0.565, SE = 0.067, *p* < 0.001, and higher log odds of selecting eligibility-related rather than relationship-sustaining attributes, b = 0.689, SE = 0.078, *p* < 0.001. The two gender-by-domain terms were jointly significant, Wald χ^2^(2) = 233.540, *p* < 0.001, supporting Hypothesis 2.

**Figure 2:**
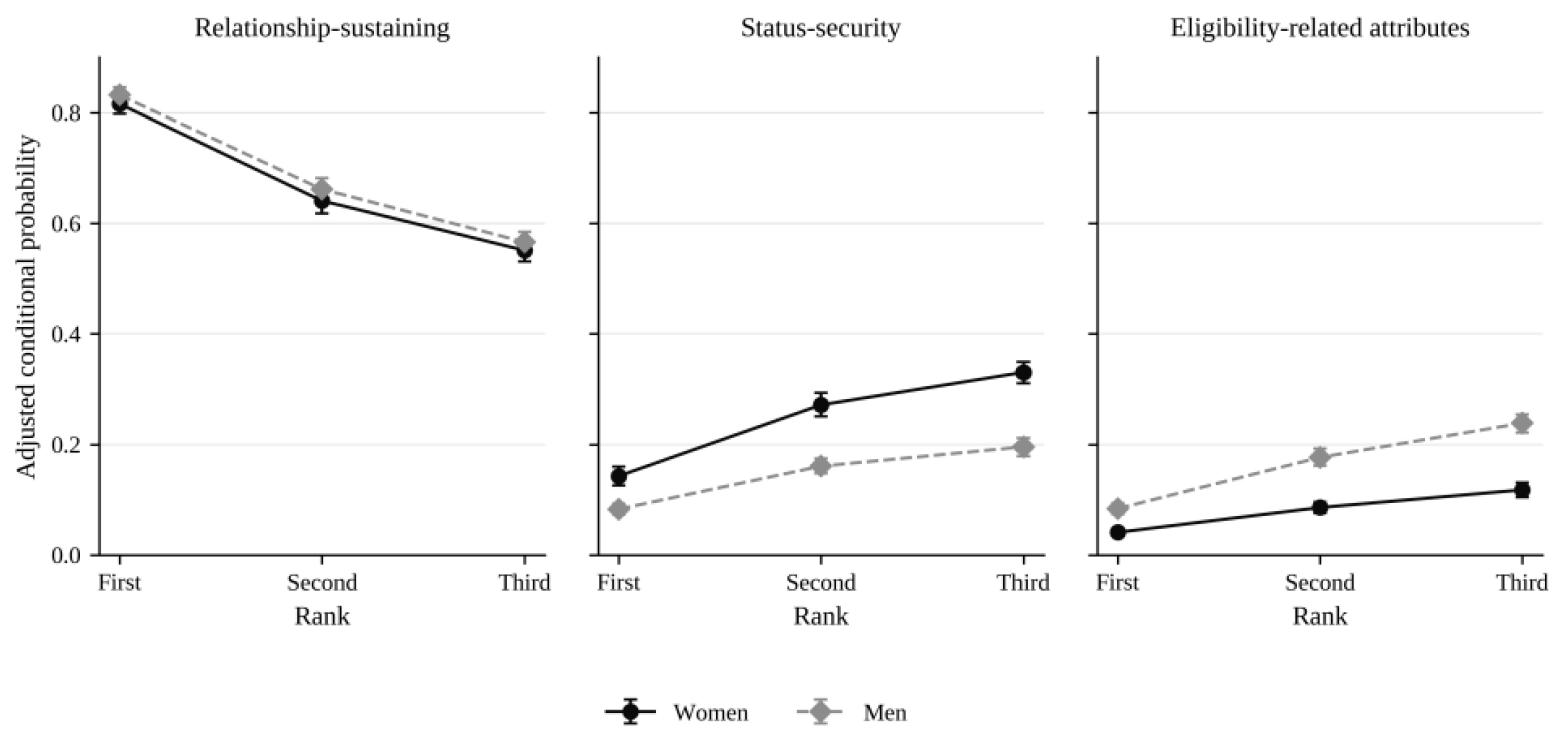
Adjusted conditional domain probabilities by gender and rank. Note. Points are standardized conditional probabilities from the fully adjusted exploded rank-ordered logit model; error bars are 95% confidence intervals. Probabilities at the second and third ranks are conditional on the observed shrinking choice sets. Gender is set counterfactually while other covariates and observed remaining alternatives are retained. N = 4704 students.

Adjusted conditional probabilities illustrate the magnitude of these differences (Figure 2; Supplementary Table S5). Women and men showed similar probabilities of selecting relationship-sustaining qualities at each rank: 0.816 versus 0.832 at the first rank, 0.641 versus 0.662 at the second, and 0.551 versus 0.566 at the third. By contrast, women had higher probabilities of selecting status-security attributes (0.143 vs. 0.083, 0.272 vs. 0.161, and 0.331 vs. 0.196), whereas men had higher probabilities of selecting eligibility-related attributes (0.084 vs. 0.041, 0.177 vs. 0.086, and 0.239 vs. 0.118). The corresponding gender differences were 6.0, 11.1, and 13.5 percentage points for status-security attributes and 4.3, 9.1, and 12.1 percentage points for eligibility-related attributes across the three ranks. These larger probability differences at later ranks reflect the changing composition of the remaining choice sets rather than stronger gender effects across ranks, because the gender-by-domain coefficients were constrained to be constant.

The larger probability gaps at the second and third ranks do not indicate that the gender association itself increased across ranks. Because the gender-by-domain coefficients were constrained to be constant, the rank-specific gaps reflect the same estimated gender contrast within progressively smaller choice sets.

Because the analytical domains, particularly the eligibility-related grouping, contain heterogeneous characteristics, we also estimated a flexible Gender × Item model (Supplementary Table S11). The gender-by-item interactions were jointly significant, Wald χ^2^(16) = 826.002, *p* < 0.001, indicating substantial variation in gender differences across individual partner characteristics. Health showed little evidence of a gender difference. Its gender interaction relative to shared values and ideals was b = −0.196, SE = 0.102, *p* = 0.053, and the adjusted men-minus-women probability contrasts were nonsignificant at the first (Δ = −0.0067, *p* = 0.084), second (Δ = −0.0064, *p* = 0.117), and third ranks (Δ = −0.0070, *p* = 0.113). By contrast, appearance and bearing showed substantially higher adjusted probabilities among men at all three ranks, with differences of 0.036, 0.076, and 0.097, respectively (all *p* < 0.001). Romantic history and age also showed positive gender contrasts, whereas ethnicity and region did not (Supplementary Table S12). These item-level results qualify the domain-level pattern: gender differentiation within the eligibility-related grouping is concentrated in particular characteristics, especially appearance and bearing, rather than being distributed uniformly across the domain.

### 3.3. Family Resources, University Tier, and Individual Orientations

With respect to Research Question 1, family socioeconomic status was positively associated with status-security priorities. A one-standard-deviation increase in family SES was associated with higher log odds of selecting status-security rather than relationship-sustaining attributes, b = 0.105, SE = 0.030, *p* < 0.001. The corresponding association with eligibility-related attributes was small and nonsignificant, b = 0.034, SE = 0.031. The family-SES-by-domain terms were jointly significant, Wald χ^2^(2) = 13.408, *p* = 0.001. By contrast, elite-university attendance was not independently associated with either status-security attributes, b = −0.122, SE = 0.083, or eligibility-related attributes, b = 0.044, SE = 0.063; the omnibus test was nonsignificant, *p* = 0.234. Thus, family socioeconomic status, but not university tier, was associated with the relative weighting of partner-preference domains.

Gender-role egalitarianism was associated with greater relative priority for relationship-sustaining qualities. A one-standard-deviation increase in egalitarianism was associated with lower log odds of selecting status-security attributes, b = −0.344, SE = 0.036, and eligibility-related attributes, b = −0.226, SE = 0.038, relative to relationship-sustaining qualities. Both coefficients were significant at *p* < 0.001, and the two egalitarianism-by-domain terms were jointly significant, Wald χ^2^(2) = 100.772, *p* < 0.001, supporting Hypothesis 3.

For Research Question 2, the moderation analysis provided no evidence that the association between gender-role egalitarianism and preference-domain priorities differed systematically between women and men. The gender-by-egalitarianism interaction was nonsignificant for status-security attributes, b = −0.103, SE = 0.075, *p* = 0.171, and for eligibility-related attributes, b = −0.015, SE = 0.072, *p* = 0.833. The two interaction terms were also nonsignificant when tested jointly, Wald χ^2^(2) = 1.955, *p* = 0.376 (Supplementary Table S10).

Research Question 3 concerned current romantic involvement. Students in romantic relationships had lower log odds of selecting status-security attributes, b = −0.278, SE = 0.065, and eligibility-related attributes, b = −0.455, SE = 0.075, relative to relationship-sustaining qualities. Both coefficients were significant at *p* < 0.001, and the omnibus test was significant, Wald χ^2^(2) = 40.657, *p* < 0.001. Romantic involvement was therefore associated with greater relative priority for relationship-sustaining qualities (Supplementary Table S4).

Among the covariates, students in their third year or above and those from county or city backgrounds placed greater relative priority on status-security attributes. Students in science, engineering, agriculture, or medicine placed less relative priority on status-security attributes than those in humanities, social sciences, arts, or management. None of these covariates was significantly associated with eligibility-related attributes.

### 3.4. Sensitivity Analyses

The association between family socioeconomic status and status-security attributes was robust across alternative SES specifications. The coefficients ranged from 0.099 to 0.139 and were statistically significant in each model, whereas associations with eligibility-related attributes were smaller and mostly nonsignificant (Supplementary Table S8). Estimates for rank, gender, gender-role egalitarianism, and romantic involvement changed little across these specifications.

A separate 15-item analysis excluded respondents whose rankings included ethnicity or region (N = 4582; Supplementary Table S9). The rank-by-domain coefficients remained positive for both status-security and eligibility-related attributes. Men continued to place less relative emphasis on status-security and greater emphasis on eligibility-related attributes, while higher gender-role egalitarianism and current romantic involvement remained associated with greater relative priority for relationship-sustaining qualities. Excluding these two rarely selected eligibility-related items did not materially alter the substantive pattern of results.

Health was also reclassified from the relationship-sustaining domain to the eligibility-related domain (Supplementary Table S13). Under this alternative classification, relationship-sustaining qualities still constituted the largest domain at the first (78.1%), second (60.1%), and third ranks (51.8%). The principal associations were preserved. Men remained less likely to prioritize status-security attributes, b = −0.600, SE = 0.068, *p* < 0.001, and more likely to prioritize eligibility-related attributes, b = 0.364, SE = 0.065, *p* < 0.001. Greater gender-role egalitarianism also remained associated with lower relative priority for both status-security, b = −0.357, SE = 0.037, *p* < 0.001, and eligibility-related attributes, b = −0.217, SE = 0.033, *p* < 0.001. The gender-by-domain and egalitarianism-by-domain omnibus tests remained significant (both *p* < 0.001). These results indicate that the principal findings were robust to the alternative placement of health.

## 4. Discussion

When Chinese undergraduates ranked three partner characteristics from 17 alternatives, relationship-sustaining qualities occupied the highest positions. Shared values and ideals and personality and temperament were especially prominent at the first rank, whereas status-security and eligibility-related characteristics appeared more often later in the ordering. Women and men showed substantial agreement at the relational top of the ranking but differed more clearly in socioeconomic and selected personal criteria. This coexistence of relational consensus and patterned differentiation is the study’s central contribution: gender differences need not imply fundamentally different partner ideals when they are concentrated in particular parts of an ordered preference structure.

Classical value theory helps clarify how partner preferences may acquire evaluative force. Values provide standards for judging desirable ends and can shape the valence attached to alternatives (Allport et al., 1960; Rokeach, 1973; Feather, 1992). In mate selection, personal values may therefore be one basis on which partner characteristics are evaluated and ordered: individuals who differ in the importance they attach to economic security, achievement, reciprocity, or concern for others may also differ in the priority they assign to socioeconomic, relational, and other partner qualities. The present study does not measure general personal values and cannot test that mechanism directly. What the ranking captures is the evaluative ordering of stated partner criteria within the specific context of partner evaluation. This distinction suggests a clear next step for research: combining validated measures of personal values with constrained partner-preference tasks would allow researchers to test whether value orientations predict the ordering of ideal-partner characteristics and whether those links vary across social contexts.

The gender results also show why domain-level findings should be interpreted alongside individual characteristics. Men were less likely than women to select status-security attributes relative to relationship-sustaining qualities and more likely to select eligibility-related attributes. Within the latter grouping, however, gender differentiation was far from uniform. Appearance and bearing accounted for the largest adjusted difference, with smaller positive contrasts for romantic history and age, whereas ethnicity and region showed no significant gender differences. Health likewise showed little evidence of an adjusted gender difference and reclassifying it as eligibility-related did not materially change the principal findings. The eligibility-related domain is therefore best understood as an analytical summary of heterogeneous criteria rather than as evidence of a single underlying psychological dimension.

Interpretation of these gender differences also requires caution. The survey recorded respondents as women or men but did not measure sexual orientation or the gender of the partner they had in mind. The present results therefore cannot establish that each respondent was evaluating an opposite-gender partner. Explanations involving economic provision, care responsibilities, fertility, or standards of physical attractiveness should be understood as features of the surrounding gendered social context rather than as directly observed heterosexual mechanisms operating for every respondent.

Family socioeconomic status was associated more selectively with partner priorities. Students from more advantaged families placed greater relative emphasis on status-security attributes, and this association remained positive across alternative SES specifications. One interpretation is that family resources shape expectations concerning education, occupation, housing, and economic compatibility (Hu & Wu, 2019; Tang & Wu, 2026). The finding does not imply that students deliberately pursue class closure or that stated preferences translate directly into assortative unions. It shows more modestly that socioeconomic background is associated with the position assigned to status-related partner criteria. Elite-university attendance, by contrast, had no independent association once family and individual characteristics were taken into account.

Gender-role egalitarianism was independently associated with greater emphasis on relationship-sustaining qualities. This distinction is theoretically important because respondent gender and gender-role attitudes capture different aspects of partner evaluation. Gender places respondents within a gendered social structure, whereas the egalitarianism measure reflects their own views about economic provision, paid work, care, and domestic responsibility. Social-role theory suggests that expectations about partners can be shaped both by the division of labor and by beliefs about those arrangements (Eagly & Wood, 1999). Consistent with this distinction, the gender coefficients remained substantial after egalitarianism was controlled, while egalitarianism remained strongly associated with relational priorities after respondent gender was included. The nonsignificant Gender × Egalitarianism × domain interaction further indicates that the association between egalitarianism and partner priorities did not differ systematically between women and men. Gender and gender-role attitudes thus represent related but empirically distinguishable sources of variation in partner evaluation.

Current romantic involvement was also associated with greater emphasis on relationship-sustaining qualities. Relationship experience may make communication, compromise, and everyday compatibility more salient, but the cross-sectional design cannot establish the direction of this association. Romantic relationships may reshape partner ideals, or individuals who already attach greater importance to relational compatibility may be more likely to enter or maintain relationships. Previous research likewise suggests that ideal-partner preferences can change across relationship transitions (Gerlach et al., 2019; Driebe et al., 2024; Hara & Yu, 2025).

The ranked format adds a different type of information from that provided by conventional importance ratings. Separate ratings identify characteristics respondents regard as desirable; constrained allocations reveal which traits are protected when resources are limited; vignette experiments estimate trade-offs among concrete partner profiles; and behavioral data record choices within actual opportunity structures (Li et al., 2002; Lewis, 2016; Yu et al., 2026). A top-three ranking instead identifies the relative position of stated criteria within a restricted ordering. It does not reproduce an actual dating market, nor does it reveal how respondents would respond to a particular person. Its value lies in showing which considerations are placed ahead of others when respondents cannot rank every desirable characteristic equally highly.

The findings also complicate a simple contrast between romantic and pragmatic partner ideals. Emotional compatibility, family resources, housing, educational prospects, and gendered responsibilities can matter simultaneously while occupying different positions in the same ordering. Placing relationship-sustaining qualities first does not imply that socioeconomic or appearance-related criteria are irrelevant; rather, these considerations tend to enter at different points in the stated preference structure.

The present data cannot establish whether the same ordering would appear in Russia, Western Europe, the Americas, or other cultural settings. Cross-cultural research nonetheless suggests reasons to expect both continuity and variation. Gender differences in the relative weight assigned to physical attractiveness and economic prospects have been observed across many societies (Buss, 1989; Thomas et al., 2020), while social-structural research indicates that the magnitude of some gender differences varies with institutional arrangements and national levels of gender equality (Eagly & Wood, 1999; Zentner & Mitura, 2012). Comparative studies using the same constrained ranking task would therefore be especially useful. Such research could test whether a relational core occupies the top of partner-preference hierarchies across societies, and whether socioeconomic, appearance-related, and background criteria enter the ordering differently under distinct institutional and cultural conditions.

### Limitations and Future Research

Several limitations qualify the interpretation of these findings. First, the ranking task captures stated partner ideals rather than attraction to particular individuals, dating behavior, or eventual partner choice. This is a substantive distinction. Some preferences may be easier to identify and articulate prospectively than others. Shared values, responsibility, and personality provide familiar ways of describing a desirable long-term partner, whereas physical attraction may depend more strongly on a particular person’s appearance, voice, manner, conversation, and interpersonal chemistry. Stated rankings may therefore reflect both what respondents value and what they can readily identify and express as important. A first-ranked characteristic should not necessarily be interpreted as exerting the greatest influence when respondents encounter an actual potential partner (Gerlach et al., 2019; Eastwick et al., 2023).

Second, selecting only three characteristics identifies relative position among stated priorities but leaves the absolute importance of unselected characteristics unknown. Rarely ranked attributes may still operate as threshold conditions or minimum requirements. The three analytical domains are also internally heterogeneous, particularly the eligibility-related grouping. Appearance and bearing accounted for substantially more selections than ethnicity or region, and the item-specific analyses showed that the characteristics within this domain did not share a uniform gender pattern. Excluding ethnicity and region, and separately reclassifying health as eligibility-related, left the principal findings substantively unchanged. These sensitivity analyses indicate that the main results are not driven by those particular classification decisions, but they do not establish the domains as latent psychological constructs. Research designed prospectively around validated multidimensional measures could examine the structure and coherence of partner-preference domains more directly. The theoretical role of the ISM is likewise limited: the PSCUS criteria were not designed to measure its three original dimensions, and the analytical domains should not be interpreted as validated ISM constructs. Classical value theory is used here to frame ranking as evaluative ordering among desirable alternatives; respondents’ general personal values were not measured directly. Future studies that pair validated ISM and personal-value measures with ranked-choice tasks could test these relationships more directly.

Third, the cross-sectional design precludes causal inference. Associations involving family socioeconomic status, gender-role attitudes, and romantic involvement may reflect selection, prior experiences, or other unmeasured processes. Longitudinal studies that repeat preference rankings across relationship transitions could clarify how partner ideals change and whether changes in relationships precede or follow changes in stated priorities.

Finally, the sample consists of undergraduates from 12 Chinese universities and the findings should not be generalized to vocational students, postgraduate students, nonstudents, or young adults in China as a whole. Gender was recorded only as woman or man, and the survey did not measure sexual orientation or the gender of the desired partner. Future research with more diverse populations and more inclusive measures of gender and partner orientation would help establish the scope of the observed patterns. Combining repeated preference measures with information about dating experiences and actual partner characteristics would also make it possible to assess how closely stated preference hierarchies correspond to subsequent relationship choices.

## 5. Conclusions

For most Chinese undergraduates, the highest-ranked partner qualities were relational rather than economic, appearance-related, or demographic. Shared values and ideals, personality and temperament, responsibility, and other relationship-sustaining qualities occupied the top of the ordering for both women and men. Gender differences were concentrated elsewhere: women assigned greater relative priority to status-security attributes, whereas men assigned greater relative priority to a heterogeneous set of eligibility-related characteristics, particularly appearance and bearing. Family socioeconomic advantage was associated with stronger status-security priorities, while more egalitarian gender-role attitudes and current romantic involvement were associated with greater emphasis on relationship-sustaining qualities.

These findings show that substantial relational consensus can coexist with socially patterned differentiation within the same preference structure. Women and men may agree on the qualities they place at the top while differing in the relative weight assigned to socioeconomic and personal criteria lower in the ordering. Ranked-choice data make this distinction visible by identifying where agreement is concentrated and where differentiation enters, rather than treating partner preferences as a set of independently rated traits.

Whether the same ordering appears in other cultural and institutional settings remains an empirical question. More broadly, the study identifies a testable connection between values and mate preferences without treating the rankings themselves as a general value measure. Personal values may help shape how individuals order relational, socioeconomic, and other partner qualities, but that mechanism must be tested with direct measures of value orientations. Future research that combines validated value measures with constrained rankings could examine this relationship explicitly. Comparative research using similar constrained-ranking designs could examine how family involvement, economic inequality, gender-role arrangements, and dating institutions shape the relative position of socioeconomic and personal partner criteria across societies.

## Figures and Tables

**Table 1:** Descriptive statistics for the continuous variables.

Variable	M	SD	Min.	Max.
Gender-role egalitarianism (6–24)	19.445	3.212	10.000	24.000
Family SES principal-component score (z)	−0.001	0.992	−3.124	2.865

Note. N = 4704. Continuous variables are reported using M, SD, and the observed range; family socioeconomic status is represented by a standardized principal-component score (z).

**Table 2:** Distribution of ideal partner characteristics by rank.

Domain	Characteristic	First, n (%)	Second, n (%)	Third, n (%)	Mean Rank	SD
Relationship-sustaining	Shared values/ideals	2134 (45.4%)	682 (14.5%)	374 (8.0%)	1.448	0.694
Relationship-sustaining	Personality/temperament	974 (20.7%)	1143 (24.3%)	720 (15.3%)	1.910	0.768
Relationship-sustaining	Responsibility	324 (6.9%)	442 (9.4%)	449 (9.5%)	2.103	0.791
Relationship-sustaining	Health	204 (4.3%)	240 (5.1%)	206 (4.4%)	2.003	0.795
Relationship-sustaining	Shared interests	186 (4.0%)	369 (7.8%)	448 (9.5%)	2.261	0.751
Relationship-sustaining	Lifestyle habits	56 (1.2%)	193 (4.1%)	445 (9.5%)	2.561	0.639
Status-security	Family origin/social ties	313 (6.7%)	385 (8.2%)	467 (9.9%)	2.132	0.808
Status-security	Education	93 (2.0%)	259 (5.5%)	227 (4.8%)	2.231	0.707
Status-security	Occupation	43 (0.9%)	94 (2.0%)	125 (2.7%)	2.313	0.738
Status-security	Housing	8 (0.2%)	23 (0.5%)	33 (0.7%)	2.391	0.704
Status-security	Income	55 (1.2%)	210 (4.5%)	314 (6.7%)	2.447	0.662
Status-security	Assets	17 (0.4%)	49 (1.0%)	77 (1.6%)	2.420	0.696
Eligibility-related attributes	Appearance/bearing	222 (4.7%)	495 (10.5%)	587 (12.5%)	2.280	0.737
Eligibility-related attributes	Romantic history	25 (0.5%)	37 (0.8%)	73 (1.6%)	2.356	0.777
Eligibility-related attributes	Ethnicity	11 (0.2%)	13 (0.3%)	17 (0.4%)	2.146	0.823
Eligibility-related attributes	Region	10 (0.2%)	18 (0.4%)	56 (1.2%)	2.548	0.701
Eligibility-related attributes	Age	29 (0.6%)	52 (1.1%)	86 (1.8%)	2.341	0.759

Note. Rank columns show n (%), with percentages calculated within rank. Mean rank and SD are calculated only among respondents who selected the characteristic; unselected characteristics have no observed rank. Lower mean ranks indicate higher conditional placement among selections, not greater overall importance in the full sample. N = 4704 students.

**Table 3:** Exploded rank-ordered logit models of ideal partner preferences.

Predictor	(1) Contextual ERL	(2) Fully Adjusted ERL
**Rank structure**		
Second rank × status-security	0.587 *** (0.059)	0.608 *** (0.059)
Second rank × eligibility-related attributes	0.658 *** (0.079)	0.688 *** (0.079)
Third rank × status-security	0.725 *** (0.065)	0.756 *** (0.065)
Third rank × eligibility-related attributes	0.915 *** (0.070)	0.979 *** (0.071)
**Family socioeconomic status and university tier**		
Family SES × status-security	0.089 ** (0.030)	0.105 *** (0.030)
Family SES × eligibility-related attributes	−0.001 (0.031)	0.034 (0.031)
Elite university × status-security	−0.225 ** (0.087)	−0.122 (0.083)
Elite university × eligibility-related attributes	0.166 + (0.087)	0.044 (0.063)
**Individual characteristics**		
Man × status-security	—	−0.565 *** (0.067)
Man × eligibility-related attributes	—	0.689 *** (0.078)
Egalitarianism × status-security	—	−0.344 *** (0.036)
Egalitarianism × eligibility-related attributes	—	−0.226 *** (0.038)
Romantic involvement × status-security	—	−0.278 *** (0.065)
Romantic involvement × eligibility-related attributes	—	−0.455 *** (0.075)
**Controls**		
Third year or above × status-security	—	0.164 *** (0.049)
Third year or above × eligibility-related attributes	—	−0.026 (0.054)
County/city residence × status-security	—	0.129 * (0.062)
County/city residence × eligibility-related attributes	—	0.039 (0.066)
Science/engineering/agriculture/medicine × status-security	—	−0.203 ** (0.078)
Science/engineering/agriculture/medicine × eligibility-related attributes	—	−0.072 (0.065)
**Omnibus Wald tests, *p* value**		
Rank × domain	<0.001	<0.001
Family SES × domain	0.011	0.001
Elite university × domain	0.010	0.234
Gender × domain	—	<0.001
Egalitarianism × domain	—	<0.001
Romantic involvement × domain	—	<0.001
**Model information**		
Students	4704	4704
Universities	12	12
Academic-unit clusters	91	91
Log likelihood	−29,819.863	−29,495.965

Note. Entries are logit coefficients with cluster-robust standard errors in parentheses. Standard errors are clustered by 91 university-by-academic-unit groups. All models include item fixed effects. Relationship-sustaining qualities at the first rank are the reference category. Predictor-by-domain coefficients are constrained to be constant across ranks. + *p* < 0.10. * *p* < 0.05. ** *p* < 0.01. *** *p* < 0.001. Family SES and gender-role egalitarianism are standardized in the ERL models.

## Data Availability

The data analyzed in this study are from the 2020 Panel Study of Chinese University Students (PSCUS), administered by the Institute of Sociology, Chinese Academy of Social Sciences. The authors accessed the data as secondary users and are not permitted to publicly redistribute the dataset. Access to the data is subject to the policies and approval of the original data custodian.

## Supplementary Materials

The following supporting information can be downloaded at: https://www.mdpi.com/article/10.3390/bs16101921/s1, Table S1: Questionnaire-Form Balance for Assignment to the Ranked-Preference Module; Table S2: Sequences of Preference Domains Across the Top Three Ranks; Table S3: Full Exploded Rank-Ordered Logit Specifications; Table S4: Omnibus Wald Tests for the Fully Adjusted ERL Model; Table S5: Adjusted Conditional Domain Probabilities by Gender and Rank; Table S6: Adjusted Item-Level Probabilities by Gender and Rank; Table S7: Gender Contrasts in Adjusted Item-Level Probabilities; Table S8: Alternative Family Socioeconomic-Status Specifications; Table S9: Fifteen-Item Sensitivity Analysis Excluding Ethnicity and Region; Table S10: Gender Moderation of the Association Between Gender-Role Egalitarianism and Partner-Preference Domains; Table S11: Item-Specific Gender Heterogeneity in Ideal Partner Preferences; Table S12: Health and Other Focal Item-Level Gender Contrasts; Table S13: Sensitivity Analysis Reclassifying Health as Eligibility-Related.
