# Supplementary material for: Relational Consensus and Gendered Differentiation: The Ranked Structure of Ideal Partner Preferences Among Chinese University Students
Source: Behav Sci (Basel). 2026 Oct 16;16(10):1921. doi: 10.3390/bs16101921 (PMC13701353; doi:10.3390/bs16101921)
Supplement: Supplementary file 1 [file behavsci-16-01921-s001.zip › behavsci-4574871-supplementary.pdf]

Supplementary Materials

Relational Consensus and Gendered Differentiation: The Ranked Structure of Ideal Partner Preferences among Chinese University Students

Zhonglu Li, Jiahui Yu, and Yuanyuan Hu

Table S1. Questionnaire-Form Balance for Assignment to the Ranked-Preference Module

**Predictor**	**Logit coefficient (SE)**
Man (vs. woman)	-0.010 (0.041)
Third year or above (vs. lower year)	-0.026 (0.042)
County/city precollege residence (vs. rural/town)	0.055 (0.054)
Science/engineering/agriculture/medicine field	0.026 (0.049)
Family SES principal-component score (z)	0.003 (0.025)
Constant	0.000 (0.061)
Undergraduate respondents	9,284
Log likelihood	-6424.398
Joint p: observed characteristics	.848

Note. The outcome equals 1 for assignment to the ranked-preference questionnaire module. The model includes respondents with nonmissing data on all five predictors (N = 9,284). For field of study, humanities, social sciences, arts, and management form the reference category. Cluster-robust standard errors are in parentheses. The joint test covers gender, year of study, precollege residence, field of study, and family SES. + $p < .10$. * $p < .05$. ** $p < .01$. *** $p < .001$.

Table S2. Sequences of Preference Domains Across the Top Three Ranks

Sequence	n	%	Sequence	n	%
R-R-R	1,478	31.4%	E-R-S	48	1.0%
R-R-S	635	13.5%	S-E-R	48	1.0%
R-R-E	498	10.6%	R-E-E	46	1.0%
R-S-R	388	8.2%	E-S-R	37	0.8%
R-E-R	305	6.5%	S-S-E	32	0.7%
R-S-S	217	4.6%	E-S-S	25	0.5%
R-E-S	158	3.4%	S-E-S	24	0.5%
R-S-E	153	3.3%	E-R-E	21	0.4%
S-R-R	148	3.1%	E-E-R	17	0.4%
E-R-R	127	2.7%	E-S-E	10	0.2%
S-S-R	94	2.0%	E-E-S	8	0.2%
S-S-S	64	1.4%	S-E-E	5	0.1%
S-R-S	64	1.4%	E-E-E	4	0.1%
S-R-E	50	1.1%			

Note. R = relationship-sustaining; S = status-security; E = eligibility-related attributes. Sequences list the domains selected at the first, second, and third ranks, respectively. N = 4,704 students. Percentages may not sum to exactly 100.0% because of rounding.

Table S3. Full Exploded Rank-Ordered Logit Specifications

Predictor	(1) Rank structure	(2) Contextual ERL	(3) Fully adjusted ERL
Rank structure			
Second rank × status-security	0.585*** (0.059)	0.587*** (0.059)	0.608*** (0.059)
Second rank × eligibility-related attributes	0.658*** (0.079)	0.658*** (0.079)	0.688*** (0.079)
Third rank × status-security	0.722*** (0.065)	0.725*** (0.065)	0.756*** (0.065)
Third rank × eligibility-related attributes	0.915*** (0.070)	0.915*** (0.070)	0.979*** (0.071)
Family socioeconomic status and university tier			
Family SES × status-security	—	0.089** (0.030)	0.105*** (0.030)
Family SES × eligibility-related attributes	—	-0.001 (0.031)	0.034 (0.031)
Elite university × status-security	—	-0.225** (0.087)	-0.122 (0.083)
Elite university × eligibility-related attributes	—	0.166+ (0.087)	0.044 (0.063)
Individual characteristics			
Man × status-security	—	—	-0.565*** (0.067)
Man × eligibility-related attributes	—	—	0.689*** (0.078)
Egalitarianism × status-security	—	—	-0.344*** (0.036)
Egalitarianism × eligibility-related attributes	—	—	-0.226*** (0.038)
Romantic involvement × status-security	—	—	-0.278*** (0.065)
Romantic involvement × eligibility-related attributes	—	—	-0.455*** (0.075)

Table S3 (continued). Full Exploded Rank-Ordered Logit Specifications

Predictor	(1) Rank structure	(2) Contextual ERL	(3) Fully adjusted ERL
Controls			
Third year or above × status-security	—	—	0.164*** (0.049)
Third year or above × eligibility-related attributes	—	—	-0.026 (0.054)
County/city residence × status-security	—	—	0.129* (0.062)
County/city residence × eligibility-related attributes	—	—	0.039 (0.066)
Science/engineering/agriculture/medicine × status-security	—	—	-0.203** (0.078)
Science/engineering/agriculture/medicine × eligibility-related attributes	—	—	-0.072 (0.065)
Model information			
Students	4,704	4,704	4,704
Universities	12	12	12
Academic-unit clusters	91	91	91
Log likelihood	-29841.763	-29819.863	-29495.965

Note. Entries are logit coefficients with cluster-robust standard errors in parentheses. Standard errors are clustered by 91 university-by-academic-unit groups. All models include item fixed effects. Relationship-sustaining qualities at the first rank are the reference category. Predictor-by-domain coefficients are constrained to be constant across ranks. Family SES and gender-role egalitarianism are standardized in the ERL models. + $p < .10$. * $p < .05$. ** $p < .01$. *** $p < .001$.

Table S4. Omnibus Wald Tests for the Fully Adjusted ERL Model

**Test**	**df**	**Wald χ^2**	**p**
Rank × domain	4	312.082	< .001
Family SES × domain	2	13.408	.001
Elite university × domain	2	2.907	.234
Gender × domain	2	233.540	< .001
Egalitarianism × domain	2	100.772	< .001
Romantic involvement × domain	2	40.657	< .001

Note. Each Wald test jointly evaluates the listed set of interaction coefficients in the fully adjusted model.

Table S5. Adjusted Conditional Domain Probabilities by Gender and Rank

Gender	Domain	First rank	Second rank	Third rank
Women	Relationship-sustaining	0.816 [0.798, 0.833]	0.641 [0.618, 0.665]	0.551 [0.531, 0.572]
Women	Status-security	0.143 [0.126, 0.160]	0.272 [0.251, 0.294]	0.331 [0.311, 0.350]
Women	Eligibility-related attributes	0.041 [0.035, 0.048]	0.086 [0.076, 0.096]	0.118 [0.105, 0.131]
Men	Relationship-sustaining	0.832 [0.819, 0.846]	0.662 [0.641, 0.682]	0.566 [0.547, 0.585]
Men	Status-security	0.083 [0.073, 0.093]	0.161 [0.148, 0.175]	0.196 [0.179, 0.212]
Men	Eligibility-related attributes	0.084 [0.074, 0.094]	0.177 [0.162, 0.193]	0.239 [0.222, 0.255]

Note. Entries are standardized conditional probabilities with 95% confidence intervals in brackets. Probabilities at the second and third ranks are conditional on the observed shrinking choice sets. Gender is set counterfactually while other covariates and observed remaining alternatives are retained. N = 4,704 students. Displayed probabilities may not sum to exactly 1.000 because of rounding.

Table S6. Adjusted Item-Level Probabilities by Gender and Rank

Panel A. Women

**Domain**	**Characteristic**	**First rank**	**Second rank**	**Third rank**
Relationship-sustaining	Shared values/ideals	0.344 (0.007)	0.184 (0.005)	0.140 (0.003)
Relationship-sustaining	Personality/temperament	0.237 (0.005)	0.208 (0.005)	0.150 (0.004)
Relationship-sustaining	Responsibility	0.082 (0.003)	0.085 (0.004)	0.087 (0.003)
Relationship-sustaining	Health	0.042 (0.002)	0.045 (0.002)	0.049 (0.002)
Relationship-sustaining	Shared interests	0.066 (0.002)	0.070 (0.003)	0.074 (0.003)
Relationship-sustaining	Lifestyle habits	0.044 (0.002)	0.048 (0.002)	0.053 (0.002)
Status-security	Family origin/social ties	0.062 (0.004)	0.114 (0.005)	0.134 (0.005)
Status-security	Education	0.029 (0.002)	0.056 (0.004)	0.069 (0.004)
Status-security	Occupation	0.013 (0.001)	0.025 (0.002)	0.032 (0.002)
Status-security	Housing	0.003 (0.000)	0.006 (0.001)	0.008 (0.001)
Status-security	Income	0.029 (0.002)	0.056 (0.003)	0.070 (0.004)
Status-security	Assets	0.007 (0.001)	0.014 (0.001)	0.018 (0.002)
Eligibility-related attributes	Appearance/bearing	0.032 (0.003)	0.066 (0.004)	0.088 (0.005)
Eligibility-related attributes	Romantic history	0.003 (0.000)	0.006 (0.001)	0.009 (0.001)
Eligibility-related attributes	Ethnicity	0.001 (0.000)	0.002 (0.000)	0.003 (0.001)
Eligibility-related attributes	Region	0.002 (0.000)	0.004 (0.001)	0.006 (0.001)
Eligibility-related attributes	Age	0.004 (0.000)	0.008 (0.001)	0.012 (0.001)

Table S6 (continued). Adjusted Item-Level Probabilities by Gender and Rank

Panel B. Men

Domain	Characteristic	First rank	Second rank	Third rank
Relationship-sustaining	Shared values/ideals	0.351 (0.007)	0.190 (0.005)	0.144 (0.003)
Relationship-sustaining	Personality/temperament	0.242 (0.004)	0.215 (0.005)	0.153 (0.003)
Relationship-sustaining	Responsibility	0.084 (0.003)	0.088 (0.004)	0.089 (0.004)
Relationship-sustaining	Health	0.043 (0.002)	0.047 (0.002)	0.050 (0.002)
Relationship-sustaining	Shared interests	0.067 (0.002)	0.073 (0.003)	0.076 (0.003)
Relationship-sustaining	Lifestyle habits	0.045 (0.002)	0.050 (0.002)	0.054 (0.002)
Status-security	Family origin/social ties	0.036 (0.002)	0.068 (0.003)	0.080 (0.004)
Status-security	Education	0.017 (0.001)	0.033 (0.002)	0.041 (0.002)
Status-security	Occupation	0.008 (0.001)	0.015 (0.001)	0.019 (0.001)
Status-security	Housing	0.002 (0.000)	0.004 (0.000)	0.005 (0.001)
Status-security	Income	0.017 (0.001)	0.033 (0.002)	0.041 (0.002)
Status-security	Assets	0.004 (0.000)	0.008 (0.001)	0.010 (0.001)
Eligibility-related attributes	Appearance/bearing	0.065 (0.004)	0.136 (0.007)	0.177 (0.007)
Eligibility-related attributes	Romantic history	0.006 (0.001)	0.013 (0.001)	0.019 (0.002)
Eligibility-related attributes	Ethnicity	0.002 (0.000)	0.004 (0.001)	0.006 (0.001)
Eligibility-related attributes	Region	0.004 (0.000)	0.008 (0.001)	0.012 (0.001)
Eligibility-related attributes	Age	0.007 (0.001)	0.016 (0.001)	0.024 (0.002)

Note. Entries are adjusted conditional probabilities with standard errors in parentheses. Probabilities at the second and third ranks are conditional on the observed shrinking choice sets. Gender is set counterfactually while other covariates and observed remaining alternatives are retained. N = 4,704 students. Because item-level probabilities are rounded to three decimals, their sums can differ slightly from the domain-level probabilities in Table S5.

Table S7. Gender Contrasts in Adjusted Item-Level Probabilities

Domain	Characteristic	First rank Δ (SE)	Second rank Δ (SE)	Third rank Δ (SE)
Relationship-sustaining	Shared values/ideals	0.007+ (0.004)	0.005 (0.003)	0.004 (0.003)
Relationship-sustaining	Personality/temperament	0.005+ (0.003)	0.007 (0.004)	0.004 (0.003)
Relationship-sustaining	Responsibility	0.002+ (0.001)	0.003 (0.002)	0.002 (0.002)
Relationship-sustaining	Health	0.001+ (0.000)	0.001 (0.001)	0.001 (0.001)
Relationship-sustaining	Shared interests	0.001+ (0.001)	0.002 (0.002)	0.002 (0.002)
Relationship-sustaining	Lifestyle habits	0.001+ (0.001)	0.002 (0.001)	0.001 (0.001)
Status-security	Family origin/social ties	-0.026*** (0.003)	-0.047*** (0.004)	-0.054*** (0.004)
Status-security	Education	-0.012*** (0.002)	-0.023*** (0.003)	-0.028*** (0.003)
Status-security	Occupation	-0.005*** (0.001)	-0.010*** (0.001)	-0.013*** (0.001)
Status-security	Housing	-0.001*** (0.000)	-0.003*** (0.000)	-0.003*** (0.000)
Status-security	Income	-0.012*** (0.002)	-0.023*** (0.002)	-0.029*** (0.003)
Status-security	Assets	-0.003*** (0.000)	-0.006*** (0.001)	-0.007*** (0.001)
Eligibility-related attributes	Appearance/bearing	0.033*** (0.004)	0.070*** (0.007)	0.089*** (0.007)
Eligibility-related attributes	Romantic history	0.003*** (0.000)	0.007*** (0.001)	0.010*** (0.001)
Eligibility-related attributes	Ethnicity	0.001*** (0.000)	0.002*** (0.000)	0.003*** (0.001)
Eligibility-related attributes	Region	0.002*** (0.000)	0.004*** (0.001)	0.006*** (0.001)
Eligibility-related attributes	Age	0.004*** (0.000)	0.008*** (0.001)	0.012*** (0.001)

Note. Δ is the adjusted probability for men minus the adjusted probability for women. Positive values indicate higher probabilities among men. Standard errors are in parentheses. + $p < .10$. * $p < .05$. ** $p < .01$. *** $p < .001$. N = 4,704 students. Contrasts are calculated from unrounded predictions, so a displayed contrast need not equal the difference between the corresponding rounded values in Table S6 exactly.

Table S8. Alternative Family Socioeconomic-Status Specifications

Panel A. Rank Structure and Family SES

Predictor	(1)<br>PCA SES	(2)<br>Mean-index SES	(3)<br>Objective SES	(4)<br>SES without occupation
Rank structure				
Second rank × status-security	0.608*** (0.059)	0.608*** (0.059)	0.609*** (0.060)	0.608*** (0.059)
Second rank × eligibility-related attributes	0.688*** (0.079)	0.687*** (0.079)	0.688*** (0.079)	0.687*** (0.079)
Third rank × status-security	0.756*** (0.065)	0.756*** (0.065)	0.757*** (0.065)	0.755*** (0.065)
Third rank × eligibility-related attributes	0.979*** (0.071)	0.979*** (0.071)	0.979*** (0.071)	0.978*** (0.071)
Family socioeconomic status				
PCA family SES × status-security	0.105*** (0.030)	—	—	—
PCA family SES × eligibility-related attributes	0.034 (0.031)	—	—	—
Mean-index family SES × status-security	—	0.103*** (0.030)	—	—
Mean-index family SES × eligibility-related attributes	—	0.032 (0.031)	—	—
Objective family SES × status-security	—	—	0.139*** (0.031)	—
Objective family SES × eligibility-related attributes	—	—	0.052+ (0.030)	—
Family SES without occupation × status-security	—	—	—	0.099** (0.030)
Family SES without occupation × eligibility-related attributes	—	—	—	0.011 (0.032)

Table S8 (continued). Alternative Family Socioeconomic-Status Specifications

Panel B. University Tier and Individual Characteristics

Predictor	(1)<br>PCA SES	(2)<br>Mean-index SES	(3)<br>Objective SES	(4)<br>SES without occupation
University tier and individual characteristics				
Elite university × status-security	-0.122 (0.083)	-0.121 (0.083)	-0.142+ (0.084)	-0.119 (0.083)
Elite university × eligibility-related attributes	0.044 (0.063)	0.045 (0.063)	0.036 (0.063)	0.051 (0.064)
Man × status-security	-0.565*** (0.067)	-0.565*** (0.067)	-0.573*** (0.067)	-0.562*** (0.067)
Man × eligibility-related attributes	0.689*** (0.078)	0.690*** (0.078)	0.686*** (0.078)	0.691*** (0.078)
Egalitarianism × status-security	-0.344*** (0.036)	-0.344*** (0.036)	-0.348*** (0.036)	-0.343*** (0.036)
Egalitarianism × eligibility-related attributes	-0.226*** (0.038)	-0.226*** (0.038)	-0.228*** (0.038)	-0.224*** (0.038)
Romantic involvement × status-security	-0.278*** (0.065)	-0.278*** (0.065)	-0.279*** (0.064)	-0.277*** (0.065)
Romantic involvement × eligibility-related attributes	-0.455*** (0.075)	-0.455*** (0.075)	-0.456*** (0.075)	-0.453*** (0.076)
Model information				
Students	4,704	4,704	4,704	4,704
Academic-unit clusters	91	91	91	91
Log likelihood	-29495.965	-29496.147	-29490.930	-29496.372

Note. Entries are logit coefficients with cluster-robust standard errors in parentheses. Standard errors are clustered by 91 university-by-academic-unit groups. All models include item fixed effects, rank-by-domain terms, and predictor-by-domain interactions for university tier, gender, gender-role egalitarianism, romantic involvement, year of study, precollege residence, and academic field. Relationship-sustaining qualities at the first rank are the reference category. + p < .10. * p < .05. ** p < .01. *** p < .001.

Table S9. Fifteen-Item Sensitivity Analysis Excluding Ethnicity and Region

Predictor	(1)<br>Fifteen-item model
Rank structure	
Second rank × status-security	0.608*** (0.061)
Second rank × eligibility-related attributes	0.701*** (0.084)
Third rank × status-security	0.750*** (0.068)
Third rank × eligibility-related attributes	0.978*** (0.076)
Family socioeconomic status and university tier	
Family SES × status-security	0.111*** (0.031)
Family SES × eligibility-related attributes	0.064* (0.032)
Elite university × status-security	-0.137 (0.086)
Elite university × eligibility-related attributes	0.031 (0.069)
Individual characteristics	
Man × status-security	-0.563*** (0.070)
Man × eligibility-related attributes	0.767*** (0.079)
Egalitarianism × status-security	-0.339*** (0.037)
Egalitarianism × eligibility-related attributes	-0.235*** (0.040)
Romantic involvement × status-security	-0.279*** (0.067)
Romantic involvement × eligibility-related attributes	-0.517*** (0.075)
Controls	
Third year or above × status-security	0.162*** (0.049)
Third year or above × eligibility-related attributes	-0.023 (0.054)
County/city residence × status-security	0.125+ (0.064)
County/city residence × eligibility-related attributes	0.068 (0.069)
Science/engineering/agriculture/medicine × status-security	-0.193* (0.082)
Science/engineering/agriculture/medicine × eligibility-related attributes	-0.036 (0.067)
Model information	
Students	4,582
Academic-unit clusters	91
Log likelihood	-28119.083

Note. Entries are logit coefficients with cluster-robust standard errors in parentheses. Ethnicity and region are excluded from the eligibility-related domain, and respondents whose top-three ranking included either item are excluded, leaving N = 4,582. Standard errors are clustered by 91 university-by-academic-unit groups. All models include item fixed effects. Relationship-sustaining qualities at the first rank are the reference category. Family SES and gender-role egalitarianism are standardized in the ERL models. + $p < .10$. * $p < .05$. ** $p < .01$. *** $p < .001$.

Table S10. Gender Moderation of the Association Between Gender-Role Egalitarianism and Partner-Preference Domains

**Predictor**	**(1) Fully adjusted ERL**	**(2) Gender-moderation ERL**
Man × status-security	-0.565*** (0.067)	-0.590*** (0.066)
Man × eligibility-related attributes	0.689*** (0.078)	0.688*** (0.078)
Egalitarianism × status-security	-0.344*** (0.036)	-0.303*** (0.050)
Egalitarianism × eligibility-related attributes	-0.226*** (0.038)	-0.220*** (0.057)
Man × Egalitarianism × status-security	—	-0.103 (0.075)
Man × Egalitarianism × eligibility-related attributes	—	-0.015 (0.072)
**Model information**		
Students	4,704	4,704
Universities	12	12
Academic-unit clusters	91	91
Joint p: Gender × Egalitarianism × domain	—	.376
Log likelihood	-29495.965	-29494.008

Note. Entries are logit coefficients with cluster-robust standard errors in parentheses. Standard errors are clustered by 91 university-by-academic-unit groups. Model 1 is the fully adjusted ERL reported in the manuscript. Model 2 adds the two Gender × Egalitarianism × domain interactions; relationship-sustaining qualities are the reference domain. Both models include item fixed effects, rank-by-domain interactions, family socioeconomic status, university tier, romantic involvement, year of study, precollege residence, and academic field. Family SES and gender-role egalitarianism are standardized. The joint p value evaluates the two three-way interaction terms. + $p < .10$. * $p < .05$. ** $p < .01$. *** $p < .001$.

Table S11. Item-Specific Gender Heterogeneity in Ideal Partner Preferences

Gender × item contrast	Logit coefficient (SE)
Man × Family origin/social ties	-0.443*** (0.079)
Man × Education	-0.173+ (0.105)
Man × Occupation	-0.444** (0.136)
Man × Appearance/bearing	0.738*** (0.092)
Man × Housing	-0.664** (0.257)
Man × Income	-1.348*** (0.127)
Man × Health	-0.196+ (0.102)
Man × Personality/temperament	0.005 (0.066)
Man × Shared interests	0.848*** (0.082)
Man × Lifestyle habits	0.218* (0.090)
Man × Romantic history	0.837*** (0.215)
Man × Ethnicity	-0.278 (0.378)
Man × Region	-0.273 (0.256)
Man × Responsibility	-0.965*** (0.077)
Man × Assets	-1.105*** (0.187)
Man × Age	0.712*** (0.212)
Students	4,704
Universities	12
Academic-unit clusters	91
Joint p: Gender × Item	< .001
Log likelihood	-29212.758

Note. Entries are coefficients from an exploded rank-ordered logit model that allows respondent gender to interact with individual partner characteristics. Shared values/ideals is the reference Gender × Item interaction. The model retains the primary rank-by-domain structure, family socioeconomic status, university tier, gender-role egalitarianism, romantic involvement, year of study, precollege residence, academic field, and item fixed effects. Standard errors are clustered by 91 university-by-academic-unit groups. The Gender × Item terms are jointly significant, Wald $\chi^2(16) = 826.002$, $p < .001$. The Health interaction, relative to shared values/ideals, is marginal and not significant at the .05 level, Wald $\chi^2(1) = 3.743$, $p = .053$. + $p < .10$. * $p < .05$. ** $p < .01$. *** $p < .001$.

Table S12. Health and Other Focal Item-Level Gender Contrasts

Panel A. Observed Selection of Health by Gender

Gender	First rank	Second rank	Third rank	Any top-three rank
Women	5.1%	5.7%	4.3%	15.1%
Men	3.4%	4.4%	4.5%	12.3%

Panel B. Adjusted Men-Minus-Women Probability Contrasts from the Flexible Gender × Item Model

Characteristic	First rank Δ (SE)	Second rank Δ (SE)	Third rank Δ (SE)
Appearance/bearing	0.036*** (0.004)	0.076*** (0.006)	0.097*** (0.007)
Health	-0.007+ (0.004)	-0.006 (0.004)	-0.007 (0.004)
Romantic history	0.004*** (0.001)	0.008*** (0.002)	0.012*** (0.002)
Ethnicity	-0.000 (0.001)	-0.001 (0.001)	-0.001 (0.002)
Region	-0.001 (0.001)	-0.001 (0.001)	-0.002 (0.002)
Age	0.004*** (0.001)	0.009*** (0.002)	0.013*** (0.003)

Note. Panel A reports unadjusted percentages of respondents selecting Health at each rank and anywhere in the top-three ranking. Panel B reports standardized conditional probability differences (men minus women) from the flexible Gender × Item model in Table S11. Positive values indicate higher adjusted probabilities among men. Health did not differ significantly by gender at any rank in the adjusted contrasts (first rank $p = .084$; second rank $p = .117$; third rank $p = .113$), whereas appearance/bearing, romantic history, and age were more likely to be selected by men. Ethnicity and region showed no significant gender differences. N = 4,704 students. + $p < .10$. * $p < .05$. ** $p < .01$. *** $p < .001$.

Table S13. Sensitivity Analysis Reclassifying Health as Eligibility-Related

Panel A. Observed Domain Distribution after Reclassifying Health

Domain	First rank	Second rank	Third rank
Relationship-sustaining (Health excluded)	78.1%	60.1%	51.8%
Status-security	11.2%	21.7%	26.4%
Eligibility-related (Health included)	10.7%	18.2%	21.8%

Panel B. Exploded Rank-Ordered Logit Model under the Alternative Health Classification

Predictor	Logit coefficient (SE)
Second rank x status-security	0.618*** (0.060)
Second rank x eligibility-related	0.490*** (0.061)
Third rank x status-security	0.744*** (0.065)
Third rank x eligibility-related	0.621*** (0.064)
Family SES x status-security	0.108*** (0.031)
Family SES x eligibility-related	0.037 (0.025)
Elite university x status-security	-0.139+ (0.083)
Elite university x eligibility-related	-0.040 (0.053)
Man x status-security	-0.600*** (0.068)
Man x eligibility-related	0.364*** (0.065)
Egalitarianism x status-security	-0.357*** (0.037)
Egalitarianism x eligibility-related	-0.217*** (0.033)
Romantic involvement x status-security	-0.287*** (0.068)
Romantic involvement x eligibility-related	-0.363*** (0.063)
Upper year x status-security	0.184*** (0.050)
Upper year x eligibility-related	0.061 (0.050)
County/city residence x status-security	0.107+ (0.063)
County/city residence x eligibility-related	-0.065 (0.059)
Science/engineering/agriculture/medicine x status-security	-0.195* (0.078)
Science/engineering/agriculture/medicine x eligibility-related	-0.015 (0.051)
Students	4,704
Universities	12
Academic-unit clusters	91
Joint p: rank × domain	< .001
Joint p: family SES × domain	.001
Joint p: elite university × domain	.212
Joint p: gender × domain	< .001
Joint p: egalitarianism × domain	< .001
Joint p: romantic involvement × domain	< .001
Log likelihood	-29591.009

Note. Health is moved from the relationship-sustaining domain to the eligibility-related domain; all other item classifications are unchanged. Entries are logit coefficients with cluster-robust standard errors in parentheses. Standard errors are clustered by 91 university-by-academic-unit groups. The model otherwise matches the fully adjusted ERL specification in the manuscript and includes item fixed effects. Relationship-sustaining qualities at the first rank remain the reference category. Family SES and gender-role egalitarianism are standardized. Reclassifying Health reduces the observed share of relationship-sustaining choices, as expected, but relationship-sustaining qualities remain the largest domain at all three ranks, and the principal gender, family-SES, egalitarianism, and romantic-involvement associations retain their original directions and statistical significance. + $p < .10$. * $p < .05$. ** $p < .01$. *** $p < .001$.
